# Supplementary material for: Strong Purifying Selection in Haploid Tissue–Specific Genes of Scots Pine Supports the Masking Theory
Source: Mol Biol Evol. 2023 Aug 11;40(8):msad183. doi: 10.1093/molbev/msad183 (PMC10457172; doi:10.1093/molbev/msad183)
Supplement: msad183_Supplementary_Data [file msad183_supplementary_data.zip › Supplementary information_Cervantes et al.docx]

|  | 0-fold sites | | | | 4-fold sites | | | |
| --- | --- | --- | --- | --- | --- | --- | --- | --- |
|  | Amount of total sites | Amount of polymorphic sites | θ_π_ | θ_W_ | Amount of total sites | Amount of polymorphic sites | θ_π_ | θ_W_ |
| Megagametophyte | 46028 | 233 | 0.0014 | 0.0015 | 10100 | 136 | 0.0036 | 0.0041 |
| Bud | 65509 | 259 | 0.0011 | 0.0012 | 14672 | 169 | 0.0034 | 0.0035 |
| Embryo | 49208 | 286 | 0.0015 | 0.0018 | 10956 | 158 | 0.0041 | 0.0043 |
| Needle | 71538 | 191 | 0.0007 | 0.0008 | 15638 | 129 | 0.0025 | 0.0025 |
| Phloem | 59316 | 203 | 0.0008 | 0.0010 | 13284 | 137 | 0.0030 | 0.0031 |
| All genes | 787707 | 2855 | 0.0009 | 0.0011 | 175736 | 1923 | 0.0030 | 0.0033 |

Table S1. Diversity estimates for tissue-specific genes and the all-genes dataset used for the estimation of Tajima’s D in Table 1. Watterson theta (θ_W_) was estimated according to Watterson 1975⁠. Theta π (θ_π_)was estimated using an implementation of the script available at https://github.com/GenTree-h2020-eu/GenTree/blob/master/kbudde/pi_from_fsfs.R. For both θ_W_ and θ_π_ we have used the fSFS downsampled at 16 alleles (20% missing data) to make use of all sites available for estimation.

|  | 0-fold | 4-fold |
| --- | --- | --- |
| Mega | 393 | 377 |
| Bud | 439 | 430 |
| Embryo | 381 | 373 |
| Needle | 633 | 621 |
| Phloem | 403 | 393 |
| All sites | 6363 | 6305 |

Table S2. Amount of putative genes used to obtain the fSFS per each tissue (Fig. 1, main text). The all-sites category includes positions located at target and off-target regions.

| Tree identification number | Used in Cervantes et al. 2021 |
| --- | --- |
| 192 | No |
| 203 | No |
| 228 | No |
| 251 | No |
| 259 | No |
| 2 | No |
| 301 | No |
| 309 | No |
| 375 | No |
| 382 | No |
| 390 | No |
| 397 | Yes |
| 443 | Yes |
| 445 | No |
| 469 | No |
| 485 | Yes |
| 494 | No |
| 498 | No |
| 520 | No |
| 95 | No |

Table S3. Identification number for the trees at Punkaharju ISS used at this study indicating if they were used for RNA sequencing in Ojeda et al 2020 and Cervantes et al 2021.


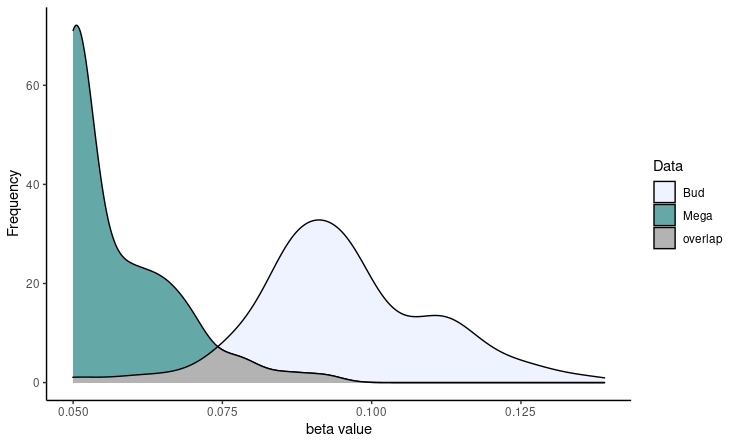


Supplementary figure 1. Density distribution of the β parameter values for the bud and megagametophyte tissue-specific gene sets and the all-sites showing the overlap area.
